# Nanopore sequencing in non-human forensic genetics

**DOI:** 10.1042/ETLS20200287

**Published:** 2021-05-18

**Authors:** Rob Ogden, Nina Vasiljevic, Stefan Prost

**Affiliations:** 1 Royal (Dick) School of Veterinary Studies and the Roslin Institute, University of Edinburgh, Edinburgh EH25 9RG, U.K.; 2 TRACE Wildlife Forensics Network, Edinburgh EH12 6LE, U.K.; 3 Institute of Forensic Medicine Zurich, University of Zurich, Winterthurerstrasse 190/52, 8057 Zurich, Switzerland; 4 Natural History Museum Vienna, Central Research Laboratories, Burgring 7, 1010 Vienna, Austria; 5 South African National Biodiversity Institute, National Zoological Garden, Pretoria 0184, South Africa

**Keywords:** minion, species identification, wildlife forensics

## Abstract

The past decade has seen a rapid expansion of non-human forensic genetics coinciding with the development of 2nd and 3rd generation DNA sequencing technologies. Nanopore sequencing is one such technology that offers massively parallel sequencing at a fraction of the capital cost of other sequencing platforms. The application of nanopore sequencing to species identification has already been widely demonstrated in biomonitoring studies and has significant potential for non-human forensic casework, particularly in the area of wildlife forensics. This review examines nanopore sequencing technology and assesses its potential applications, advantages and drawbacks for use in non-human forensics, alongside other next-generation sequencing platforms and as a possible replacement to Sanger sequencing. We assess the specific challenges of sequence error rate and the standardisation of consensus sequence production, before discussing recent progress in the validation of nanopore sequencing for use in forensic casework. We conclude that nanopore sequencing may be able to play a considerable role in the future of non-human forensic genetics, especially for applications to wildlife law enforcement within emerging forensic laboratories.

## Introduction

Since its inception in the late 1980s, the development of forensic genetics has been characterised by periods of rapid technological advancement, followed by consolidation and the establishment of validated methods for the routine production of DNA evidence. Genetic markers for individualisation evolved from multi-locus probes, to single-locus probes and then to STR (short tandem repeat) markers and SNPs (single nucleotide polymorphisms). In parallel, DNA sequencing has been used to characterise lineage or species diversity for application to both human and non-human evidence identification. DNA sequencing has traditionally targeted mitochondrial DNA gene regions through the use of the Sanger sequencing method. However, the advent of 2nd and 3nd generation sequencing methods is gradually leading to the transfer of forensic genetic analysis onto high-throughput sequencing (HTS) platforms, for both individualisation and higher-level taxonomic assignment.

This latest phase of technological change has coincided with the rapid expansion of *wildlife DNA forensics*, the application of forensic genetic analysis to the identification of non-human biological evidence. Wildlife DNA forensics is a component of forensic genetics, characterised by the breadth of animal and plant taxa present in evidence samples and the specialist evolutionary and population genetic knowledge required to interpret analytical results [1]. It is also often most needed in biodiversity-rich countries typically lacking substantial scientific infrastructure. Taken collectively, this means that there is significant interest in the availability of low-cost, robust DNA sequencing systems within the global wildlife forensic community. Against this background, this review focuses on the potential benefits and limitations of using *nanopore sequencing* for non-human forensic genetic analysis.

## Nanopore sequencing technology

**Figure 1.**
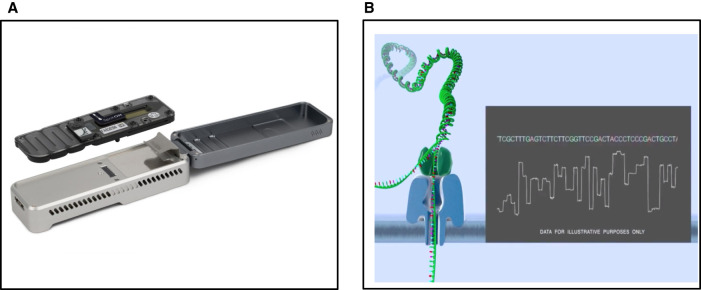
MinION sequencing. (**A**) Oxford Nanopore Technologies’ MinION device and flow cell. (**B**) Nanopore sequencing process: An ionic current is passed through a nanopore by setting a voltage across the membrane. Single-stranded DNA is funneled through the pore with the help of a motor protein. The current changes depending on the motif of the base motifs that pass through the pore (in 6 bps increments).

Even though the concept of nanopore-based sequencing has been around since the mid 90's see [2], it took until 2014 for the first commercial nanopore-based sequencer to be available — Oxford Nanopore Technology's MinION [3]. At a size of 10 × 3.2 × 2 cm and 90 g, and powered via a USB cable, ONT's MinION is the smallest sequencer on the market (Figure 1A). At the heart of its technology are small biological pores (so-called nanopores) embedded into a membrane in the sequencing flow cell. Each nanopore channel is controlled and measured by an application-specific integrated circuit (ASIC). When single-stranded DNA (ssDNA) passes through a channel, the changes in ionic current (which progresses unidirectional from one side of the membrane to the other) are measured at a contraction in the pore, called the reader-head (Figure 1B). Depending on the sequence of the nucleotides that travel through the nanopore, these current changes show specific patterns, which can later be transferred into nucleotide sequences (via a process called base-calling). To funnel the DNA through a nanopore, sequencing adapters need to be attached to the double-stranded DNA (dsDNA). These sequencing adapters include the motor protein, that unwinds the dsDNA and passes the now ssDNA through the nanopore. The flow speed through the pore can vary substantially [4], which makes the identification of the exact number of nucleotides in homopolymeric regions difficult (a systematic error of the MinION). To improve the accuracy of the sequencing in the new generation of pores (R10.x), the ssDNA passes through two reader-heads in an extended barrel; this has substantially reduced error rates caused by homopolymeric regions [5]. In a single run, ONT's MinION (for a maximum run time of 48 h, with up to 512 nanopore channels) can produce up to 44 Gb output with a standard flow cell and up to 2 Gb with the so-called flongle flow cell. Beside the USB-sized MinION, ONT also offers the GridION, which is a platform that can run 5 MinION flow cells in parallel, and the PromethION, which can house either up to 24 or 48 PromethION specific flow cells (https://nanoporetech.com/products/comparison#platform), with up to 3000 nanopore channels. Each of these PromethION specific flow cells can produce up to 200 Gb in sequence output.

## Applications to species identification

Nanopore-based sequencing is used in a variety of both DNA and RNA sequencing applications. While its long-read length (of up to ∼1 Mb, [6]) makes it very attractive for the generation of *de novo* genome assemblies (see e.g. [6,7]), its low upfront costs (∼1000 USD) and its portability offer huge advantages for DNA- or meta-barcoding experiments in countries with limited infrastructure and funds for molecular biomonitoring [8–12], and for teaching and local capacity building [13,14]. The use of the MinION has been extensively investigated for biodiversity research and biomonitoring projects (reviewed in [15]). Furthermore, within conservation biology, Seah et al. [12] have successfully shown that it is possible to generate accurate species-diagnostic DNA barcode sequences from preserved and non-invasively collected wildlife samples (scat, hair, feather, fresh frozen liver, and FFPE liver). Apart from its low upfront and running costs, and its portability, a huge advantage is its capability for multiplexing multiple samples on a single flow cell. While ONT offers indexing kits for up to 96 samples at the moment, custom indexing using either single- (same index on both ends of the DNA sequence) or double-indexing (different indexes on each end) allow for the pooling of hundreds to thousands of samples on a single flow cell [16].

There are a variety of tools and pipelines published for the analysis of DNA barcoding data generated with the MinION sequencer (e.g. Consesion [17], ONTrack [18], SAIGA [12], NGSpeciesID [19]). In the first step of most applications using MinION data, the raw signal from the sequencer has to be transformed into nucleotide sequences. During the base-calling, the raw signal (in fast5 format) is transferred into nucleotide sequences and corresponding quality scores (in fastq format), so-called Phred scores. The Phred score is the likelihood that a given base in a sequence is wrongly called. A Phred score of 20 indicates a 1% chance of this base being wrong, while a Phred score of 30 indicates a 0.1% chance. Next, the individual reads are usually clustered according to their similarity [20], and consensus sequences for the major cluster/s generated. Finally, the consensus sequence accuracy is further improved using consensus polishing. In this step the individual reads are usually mapped back to the consensus sequence, and basepair errors are corrected using information from the multiple mapping reads (e.g. Medaka (https://github.com/nanoporetech/Medaka) or Racon [21]) or using additional information from the raw fast5 data (e.g. Nanopolish (https://github.com/jts/nanopolish). Species identification via DNA barcoding on the MinION sequencer has successfully been carried out for a variety of animal, fungi and plant taxa (e.g. [9,12,16,17]). An often-overlooked advantage of the MinION sequencer when it comes to DNA barcoding, is its long-read length. Several studies have shown that longer DNA barcodes can show improved species identification capabilities and strongly increased phylogenetic resolutions ([15,17], etc.). These characteristics establish the potential for using nanopore sequencing in wildlife forensic science.

## Constraints on nanopore sequencing for forensics

The field of forensics genetics requires reliable, reproducible and highly accurate DNA sequencing methods. At the same time, in comparison with mainstream academic molecular biology research, DNA sequencing methods should work with highly degraded, low-level DNA, and cope with the presence of biological contaminants or sample mixtures. Conventional Sanger sequencing is still the gold standard method in forensic genetics. It continues to produce highly accurate and reliable sequences with an established sequencing accuracy of 99.999% [22]. However, Sanger sequencing has limitations; it can generate only a single read per amplified PCR product (amplicon) and is unable to analyse mixed or contaminated DNA samples consisting of two or more donors [23].

To overcome these constraints, the forensic community has recently started turning to HTS technologies which are capable of producing millions of reads from hundreds of samples, in parallel, which can efficiently distinguish contamination or contributions from multiple biological sources in mixtures. For example, the prominent MiSeq platform from Illumina has been validated for use in human STR profiling [24] and for the identification of endangered species in mixed samples for non-human forensic purposes [25]. Illumina sequencing is massively parallel and highly accurate (99.99%), delivering comparable sequence read quality to Sanger sequencing. Despite their advantages, platforms such as the MiSeq are expensive. Furthermore, for generating the very small amounts of data required for amplicon resequencing, data production is cost-effective only when large numbers of samples are sequenced at the same time. This makes MiSeq impractical and beyond the reach of many low-throughput end-users, such as non-human forensic scientists. New emerging HTS technologies such as ONT's MinION have the potential to address this limitation by delivering cost-effective data on an affordable platform for forensic purposes.

In terms of sensitivity to input DNA amount and quality, nanopore sequencing performs well on low levels of input DNA as it can easily be used to sequence libraries generated through PCR amplification of standard forensic DNA markers. While capable of very long-read sequence generation (up to 10^6^ bases per read), nanopore sequencing is also effective for short amplicon sequencing down to ∼200 bp bases (the required minimum read length of the ONT platforms; [26]). Probably the major limitation of nanopore sequencing is its lower read accuracy (currently ∼5–15%; [27]) when compared with the highly accurate sequencing methods of Sanger and Illumina. Although the level of MinION sequence error continues to decrease with successive updates to sequencing chemistry and improvement of bioinformatic tools, it is still common to encounter a situation where the nucleotide sequence result generated by taking the majority consensus of multiple MinION reads at each base, differs from every individual raw sequence read observed. Despite this, the generation of highly reliable consensus sequences has been shown for many applications such as genome or chromosome assemblies (e.g. [6,7]), DNA barcoding [9,26,28], HLA typing [5], and meta-barcoding [11].

## Data processing and bioinformatic considerations

From a forensic perspective, it is important to consider the way in which raw genetic data is treated to generate an evidential result that can be interpreted by the court. Any requirement for highly complex, advanced bioinformatic processing not only restricts the number of forensic practitioners capable of employing the technique, but may also render the evidence unusable by the prosecution, if it is not possible to communicate the analytical process effectively in the courtroom. This is a challenge for all HTS technologies. To produce a single DNA sequence result from nanopore output data it must be passed through two basic stages: raw data processing and consensus sequence generation.

**Figure 2.**
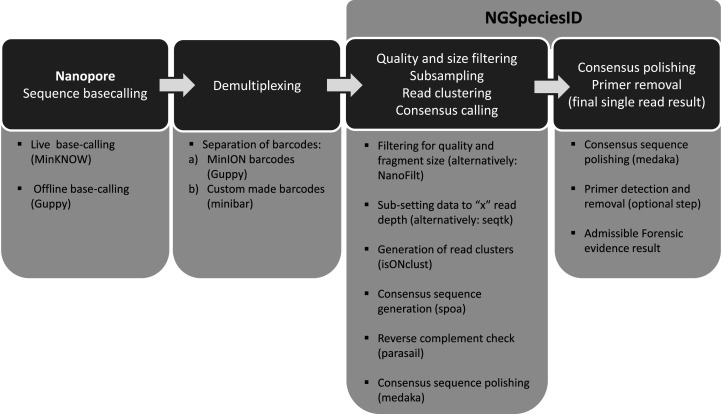
Bioinformatic analysis. Flow diagram of nanopore data analysis pipeline showing initial data processing (base-calling and demultiplexing) followed by bioinformatic analysis using the NGSpeciesID software to generate a single consensus sequence for subsequent species identification.

During raw data processing, base-calling algorithms are used to determine the actual DNA sequence within each read, followed by demultiplexing of indexed samples if multiple indexes have been used. Base-calling is initially achieved using the ONT MinKNOW software, which can be performed in real time as soon as data acquisition is initiated, to produce either base-called FASTQ files or raw FAST5 (HDF5), depending on the user's preference (Figure 2). FAST5 files can also be used for offline base-calling using Guppy (Oxford Nanopore Technologies, U.K.) which is a data processing toolkit from ONT that contains base-calling algorithms and downstream analysis such as demultiplexing barcodes, adapter trimming and alignment. Alternatively, to demultiplex libraries based on ONT or custom design barcodes, tools such as *minibar* [26] are available. After demultiplexing, reads below a certain quality score can be removed with programmes such as *NanoFilt* [29] or within consensus generation software tools such as NGSpeciesID [19].

Following raw data processing and quality filtering, the sequence data itself is ready for analysis. There will be multiple (100's to 100 000's) sequence reads associated with each sample that can be processed through a range of bioinformatic pipelines currently available (depending on the user needs). The primary objective here is to create a single consensus sequence for each major species contributing to the DNA evidence; this is achieved through three common steps: read clustering, consensus sequence generation, and consensus sequence polishing (Figure 2). At this stage, low-level contaminant sequences (e.g. microbial sequence reads) can be discarded. For unmixed samples, the final result will typically be a single consensus sequence that can subsequently be used for DNA species identification through comparison to an appropriate reference database.

## Opportunities and current research in non-human forensics

Assuming that the technical challenges relating to sequence quality and data processing can be overcome, there appear to be several potential applications of nanopore sequencing to non-human forensics. The most immediate opportunity is the use of nanopore sequencing for species identification in wildlife DNA forensics. The current restrictions of capital equipment and maintenance costs faced by wildlife forensic scientists attempting to develop sequencing capacity could be largely overcome through the use of devices such as the MinION sequencer, which only costs ∼$1000 USD and has no annual maintenance requirements (Table 1). The distribution of ONT equipment for research and diagnostic purposes is already global, with logistics in place to enable delivery and basic training. Uptake of the technology and therefore establishment of operator expertise, has grown significantly in the wake of the coronavirus pandemic, with nanopore sequencing being routinely used to characterise viral variants in many countries previously lacking such capacity (see e.g. [30,31]). The successful transfer of sequence-based taxonomic identification from medical diagnostic to wildlife forensic environments is clearly possible, but requires the production of standardised protocols and formal method validation in order to demonstrate that the accuracy and robustness of the resulting data are suitable for generating courtroom evidence. Many recent studies have sought to address these needs.

**Table 1.** Comparative table of Sanger/MiSeq/Nanopore sequencing platforms for DNA species identification summarizing approximate platform cost; running cost; analysis time; data output; accuracy and key benefits/limitations

Platform	Platform cost	Running costs (per mb)	Analysis time			Data types	Read length	Output	Accuracy (consensus)	Key benefits and limitations
			Wet laboratory	Run time	Dry laboratory					
Sanger	$250 000	$500	8 h	20 min–3 h	5–10 min/per sample	ab1	<1000 bp	1 read per sequencing reaction	99.999%	Low throughput High accuracy
Illumina MiSeq	$125 000	$0.5	8–24 h	21–56 h	∼15 min/1–50 samples	fastq	2 × 300 bp	15 Gb (∼25 M reads)	99.99%	High-throughput Good accuracy Short reads
ONT MinION	$1000	$1.9–$9	8–10 h	1–48 h	∼15 min/1–50 samples	fast5, fastq	200–∼1 mb	1–44 Gb	>99%	Affordable Lower accuracy Long reads

The design and implementation of validation studies should focus on processes and parameters that have not been previously validated and for which there is the potential for inherent or user-driven variation that will influence the analytical result. The use of DNA sequencing for species identification has already been subject to validation in relation to the use of different gene regions [32,33] and methods for conducting sequence similarity searches [34]. The protocols for conducting nanopore library preparation and sequencing are published by ONT and are the subject of regular updates and revisions, as is the proprietary ONT software for initial sequence base-calling. These stages of analysis are analogous to employing the sequencing reaction and data collection software used to generate Sanger sequence data (sample chromatograms). While these steps need to work in order to successfully generate result data, there is typically no deviation from the manufacturer's protocols and where user intervention does occur, it will not alter the DNA sequences obtained, therefore, there is no obvious need to conduct validation studies of these specific steps. The primary focus of method validation has, therefore, been from the downstream processing of base-called sequence data, through to the production of a single result sequence for a given sample. These steps involve several user-defined parameters, a range of software and pipeline options and the need to make decisions on issues such as sequence read quality, error rate, read depth, and consensus sequence generation.

In contrast with the analysis of Sanger sequence data, the bioinformatic construction of consensus sequences for use with amplicon-based approaches on the MinION remains an area of active method development; several bioinformatic software solutions are available and no single tool is recognized as a standard approach. In the first validation of MinION sequencing data for molecular genetic non-human forensic species identification, Vasiljevic et al. [35] examined the NGSpeciesID software [19] for the generation of consensus sequences for single-donor samples. In its newest version, this software includes all necessary steps (such as quality and length filtering, read subsampling and clustering, consensus generation, primer removal and polishing) that are needed to generate reliable consensus sequences and is thus easier to use than other available software solutions (https://github.com/ksahlin/NGSpeciesID). The validation study assessed the impact of sequence data variation on the accuracy of species identification, measured against Sanger sequence data generated from the same samples. It included nanopore data from six species including birds and mammals and assessed NGSpeciesID performance at a range of sequence read depths (50–5000X) compared against many metrics including individual sequence read variation (error estimation), consensus sequence accuracy, divergence from Sanger sequence data and an evaluation of the impact of any observed sequence divergence on subsequent species identification. Validation results clearly demonstrated the use of nanopore data in conjunction with the NGSpeciesID method for robust species identification, strongly supporting its use in forensic genetics (Table 2).

**Table 2.** **Summary of validation study results for species identification using the ONT MinION in conjunction with the NGSpeciesID pipeline (after Vasiljevic et al.** [35]**)**.

Species	Sequence type	No. of replicates (out of 50)	% divergence from Sanger	% sim to rank 1 species	% sim to rank 2 species	Barcoding gap
Wild boar	Sanger (control)			100	97.15	2.85
	Consensus 1	50	0	100	97.15	2.85
Roe deer	Sanger (control)			100	98.30	1.9
	Consensus 1	23	0	100	98.30	1.9
	Consensus 2	27	0.24	99.76	98.10	**1**.**66**
Chamois	Sanger (control)			99.27	95.62	3.65
	Consensus 1	47	0.00	99.27	95.62	3.65
	Consensus 2	1	0.24	99.03	95.38	3.65
	Consensus 3	1	0.24	99.03	95.39	**3**.**64**
	Consensus 4	1	0.24	99.03	95.39	**3**.**64**
Lynx	Sanger (control)			100	94.92	5.08
	Consensus 1	49	0.00	100	94.92	5.08
	Consensus 2	1	0.24	99.76	94.67	5.09
Snow leopard	Sanger (control)			100	91.90	8.10
	Consensus 1	49	0.00	100	91.90	8.10
	Consensus 2	1	0.24	100	91.89	8.11
Inca tern	Sanger (control)			99.52	91.45	8.07
	Consensus 1	47	0.00	99.52	91.45	8.07
	Consensus 2	1	0.24	99.52	91.45	8.07
	Consensus 3	2	0.24	99.52	91.41	8.11

The maximum observed sequence divergence (MinION — Sanger) across 50 replicates and six species was 0.24%, or 1 base for the 420 bp mtDNA cyt b sequence; differences had no impact on nBLAST species identification result in Genbank. The barcoding gap is the difference in percent sequence similarities between the test sample-to-rank 1 species match and the test sample-to-rank 2 species match. Any reductions in the barcoding gap when using MinION sequence data, compared with Sanger sequencing, were negligible (indicated in bold).

## Future directions

The establishment of nanopore sequencing as a routine approach for wildlife DNA forensic species identification would represent a significant development in the application of this technology to forensic genetics. Beyond this specific application, it is worth considering how else nanopore sequencing might be applied. HTS is also being used for STR genotyping in human forensics [36] using larger platforms such as Illumina MiSeq. However, to date, MinION based sequencing has been shown to be of limited use for STR DNA profiling [37–39], due to the frequency of homopolymeric regions and indels in and around STRs and the very short (<200 bp) length of many STR loci. Nanopore sequencing may have the potential for SNP DNA profiling through reduced representation libraries such as genotyping-by-sequencing (GBS) approaches, which like sequence-based identification, should enable accurate characterization of individual nucleotides in a consensus sequence. The size and portability of the ONT MinION does allow nanopore sequencing to be conducted in the field, via lab-in-a-backpack systems [9], which has also led to some discussion over the idea of portable forensic solutions. Future validation studies may enable the use of portable sequencing technologies by scientific officers operating under appropriate quality control measures; however, it is likely that any such applications would be limited to presumptive testing (similar in purpose to alcohol breathalyzer tests) and as such would require a careful cost-benefit analysis. Nevertheless, the MinION's portable characteristics do create many more options for the development of low-cost laboratories which should support the ongoing decentralization of molecular genetic analysis.

Beyond casework considerations, the availability of reference genomes to support the development of wildlife DNA forensic tools is another area where affordable HTS is required. Generating comparable data from multiple individuals belonging to endangered species distributed across many countries is logistically challenging due to restrictions on sample movements and the lack of local infrastructure. The widespread availability of nanopore technology should facilitate collaborative wildlife forensic research and development projects across borders.

## Summary

- Nanopore sequencing is an established HTS technology with potential applications to non-human forensic genetics.
- One of the clearest current opportunities is the use of MinION sequencing for species identification, particularly in biodiverse countries with limited existing laboratory infrastructure.
- Challenges to the transfer of nanopore sequencing to forensics exist, particularly around levels of sequence error, however, established protocols and validation data support its use for the analysis and production of casework evidence.

## Open Access Statement

Open access for this article was enabled by the participation of University of Edinburgh in an all-inclusive *Read & Publish* pilot with Portland Press and the Biochemical Society under a transformative agreement with JISC.

## Abbreviations

dsDNA: double-stranded DNA
HTS: high-throughput sequencing
SNPs: single nucleotide polymorphisms
ssDNA: single-stranded DNA
STR: short tandem repeat
